# The Effects of Adding Walnut Green Husk on the Quality of Alfalfa Mixed Silage, Protein Degradation, Microbial Community, and Their Interrelationships

**DOI:** 10.3390/metabo16030187

**Published:** 2026-03-11

**Authors:** Naibi Abulaiti, Gulinigaer Aiyisirehong, Aibibula Yimamu

**Affiliations:** 1 Xinjiang Key Laboratory of Grassland Resources and Ecology, College of Grassland Science, Xinjiang Agricultural University, Urumqi 830052, China; xjauyzb@xjau.edu.cn; 2 Xinjiang Academy of Agricultural Sciences, Institute of Microbiology, Urumqi 830091, China; nkytsg@xaas.ac.cn

**Keywords:** alfalfa, bacterial community, protein degradation, silage quality, walnut green husk

## Abstract

**Highlights:**

**What are the main findings?**

- Adding walnut green husk (WGH) to alfalfa silage significantly improves fermentation quality, characterized by reduced organic acid content and the absence of mold.
- WGH addition linearly decreases non-protein nitrogen (NPN), protease activity, and slowly degradable protein (PB3), while linearly increasing rapidly degradable protein (PB1) and in vitro dry matter digestibility (IVDMD).
- WGH enriches the microbial community by increasing the relative abundance of beneficial *Lactiplantibacillus* and *Levilactobacillus*, which are negatively correlated with protein degradation factors.

**What are the implications of the main findings?**

- This study identifies WGH, an agricultural by-product, as an effective silage additive that mitigates protein degradation, offering a strategy to improve the utilization efficiency of alfalfa protein in ruminant production.
- The findings support a sustainable approach to waste management by recycling walnut green husks, contributing to a circular economy model in agriculture.
- The optimal inclusion level of 120 g/kg WGH provides a practical guideline for farmers to enhance silage nutritional value and reduce feed costs.

**Abstract:**

**Objectives**: This experiment was conducted to investigate the effects of adding walnut (*Juglans regia* L.) green husk (WGH) on the quality of alfalfa mixed silage, protein degradation, microbial community, and their interrelationships. **Methods**: Alfalfa (*Medicago sativa* L.) fresh grass and WGH dried powder were used as raw materials to prepare three mixed silages of alfalfa fresh grass with 80 g/kg (A1), 120 g/kg (A2), and 160 g/kg (A3) of WGH dried powder, respectively, with alfalfa fresh grass silage as the control group (CK). After 60 days of ensilage, samples were taken and analyzed, with three replicates per treatment. **Results**: WGH treatment significantly improved alfalfa silage fermentation and nutritional quality. It reduced undesirable fermentation products while promoting beneficial lactic acid bacteria and preventing mold growth. Increasing the WGH ratio enhanced dry matter content and digestibility, with only a minor effect on crude protein. These results suggest that WGH is an effective silage additive for improving both fermentation characteristics and feed value. With the increase in the proportion of WGH, the proportions of rapidly degradable protein (PB1) and medium rate degradable protein (PB2) increased linearly, while the proportions of free amino acid nitrogen (FAA-N), peptide nitrogen (Peptide-N), slow degradable protein (PB3) and binding protein (PC) decreased linearly and the protease activity decreased significantly (*p* < 0.05). Bacterial community analysis showed that the relative abundance of *Lactiplantibacillus* and *Levilactobacillus* in the silage increased after WGH was added, while the relative abundance of *Acetobacter*, *Pantoea*, *Weissella* and *Serratia* decreased. **Conclusions**: Compared with pure alfalfa silage, the addition of WGH has a positive effect on silage quality, protein degradation and bacterial community structure, and the addition of WGH with 120 g/kg is more suitable.

## 1. Introduction

Walnut (*Juglans regia* L.) green husk (WGH), the green outer layer of the Juglans regia shell, accounts for approximately 71% of the fruit’s total weight [1]. China, as the world’s largest walnut producer with an output of 5.87 million tons in 2023, generates substantial amounts of WGH as a primary by-product [2]. However, this resource is often underutilized and discarded as waste. WGH contains 13.8% crude protein and is rich in bioactive compounds, including polyphenolic tannins, naphthoquinones, flavonoids, polysaccharides, and polyphenols, which confer antioxidant and antibacterial properties [3]. Notably, polyphenolic tannins can inhibit protease activity, thereby potentially reducing protein degradation and non-protein nitrogen (NPN) production in silage. WGH has been recognized as a potential feed resource due to its bioactive properties [4]. However, its application in silage systems has not been systematically evaluated, warranting the present investigation.

Alfalfa (*Medicago sativa* L.) is a high-yielding, high-quality forage with abundant crude protein. Nevertheless, its ensiling is challenging due to its high moisture content, high buffering capacity, and low water-soluble carbohydrate content [5]. During fermentation, extensive protein degradation occurs as plant and microbial proteases convert true protein into NPN [6]. Studies have demonstrated that co-ensiling alfalfa with additives rich in active compounds can mitigate these issues. For instance, adding dried jujube powder reduced NPN content in alfalfa silage [7], while mixing with red clover (*Trifolium pratense*), which contains polyphenol oxidase, decreased protein degradation and protease activity [8]. These findings suggest that incorporating secondary products with bioactive components into alfalfa silage can enhance fermentation quality and inhibit protein breakdown.

Given its high content of polyphenolic tannins, WGH presents a promising candidate for improving alfalfa silage. However, no studies have comprehensively evaluated the effects of WGH on alfalfa silage by integrating Cornell Net Carbohydrate and Protein System (CNCPS) protein fractions, bacterial community analysis, and their interrelationships. Therefore, this study aimed to investigate the effects of WGH addition on protein metabolism, microbial community dynamics, and their interrelationships in alfalfa silage. The research was conducted during the walnut harvest season to utilize fresh WGH. The results are expected to provide a theoretical basis for enhancing the quality and protein utilization efficiency of alfalfa silage.

## 2. Materials and Methods

### 2.1. Raw Materials and Additives

WGH was collected from Fengda Agriculture and Forestry Technology Development Co., Ltd., in the Aksu Prefecture, Xinjiang, China (41°20′ N, 78°85′ E), which has a temperate continental arid climate with a mean annual temperature of approximately 10.8 °C and mean annual precipitation of 50−80 mm. The collected WGH was air-dried and ground into powder using a small grinder (Shanghai Jingxin Industrial Development Co., Ltd., Shanghai, China) for later use. Fresh alfalfa (‘Longwei 6010′) was harvested during the squaring stage of its third crop in September 2025 from an alfalfa planting area in Macaiti Village, Aktohai Township, Wushi County, Aksu Prefecture, Xinjiang, China (40°43′−41°51′ N, 78°23′−80°01′ E). This region has a warm temperate continental semi-arid climate, with a mean annual temperature of 9.4 °C, annual precipitation of 70−120 mm, and a frost-free period of 183−206 days. The harvested alfalfa was chopped into approximately 2 cm segments using a stationary forage chopper (Zhengzhou Kewei Machinery Co., Ltd., Zhengzhou, China) for ensiling. The moisture and nutrient contents of the raw materials are presented in Table 1. For the silage additive, a commercial freeze-dried powder containing *Lactobacillus plantarum*, *Lactobacillus buchneri*, *Bacillus coagulans*, and *Bacillus subtilis* (Institute of Microbial Application, Xinjiang Academy of Agricultural Sciences, Urumqi, China) was used. The viable bacterial count was 1.0 × 10^11^ CFU per gram of the product. To prepare the inoculant, 10 g of the additive was dissolved in 1 L of a 1% (*w*/*v*) brown sugar solution [9]. The mixture was stirred, activated at room temperature (25–32 °C) for 30 min, and then diluted with distilled water to achieve a final application rate of 2.0 × 10^6^ CFU per gram of fresh material. The prepared inoculant was applied evenly onto the forage materials during mixing at a rate of 2 mL per kg of fresh forage using a handheld manual sprayer (Taizhou Luqiao Jucheng Plastic Factory, Taizhou, China). Total tannin content in walnut green husk was determined according to the Chinese national standard method for tannin determination in feeds (GB/T 27985-2011 [10]). This method is based on spectrophotometric detection after extraction with acetone solution and reaction with tungstate-phosphomolybdate reagent, with absorbance measured at 760 nm.

### 2.2. Silage Preparation

Four treatments were established in this experiment: a control group (CK) consisting solely of fresh alfalfa, and three treatment groups where WGH powder was added to fresh alfalfa at inclusion rates of 80, 120, and 160 g/kg (on a fresh matter basis), designated as A1, A2, and A3, respectively.

For each treatment, the fresh alfalfa and WGH powder were thoroughly mixed according to the specified ratios. Approximately 300 g of the mixture was packed into a polyethylene vacuum bag (20 cm × 25 cm) and sealed, achieving an initial packing density of approximately 600 kg/m^3^. Each treatment was replicated three times (resulting in a total of 12 bags). All bags were stored in the dark at room temperature (25 °C) for 60 days. After the ensiling period, the bags were opened for sample collection and subsequent analysis.

### 2.3. Silage Quality Analysis

The silage quality was assessed by measuring pH, microbial counts, organic acid concentrations, dry matter (DM), crude protein (CP), and in vitro dry matter digestibility (IVDMD). For pH measurement, a 20 g sample was homogenized with 180 mL of deionized water, filtered, and the pH of the filtrate was determined after 30 min using a pH meter (Mettler-Toledo, Columbus, OH, USA) [7]. Microbial counts were analyzed after 60 days using the plate counting method. Briefly, 10 g of silage was mixed with sterile saline, shaken, and appropriate dilutions were plated in triplicate on de Man, Rogosa and Sharpe (MRS) agar for lactic acid bacteria and potato dextrose agar (PDA) for yeasts and molds, followed by incubation at 37 °C for 48 h in a microbiological incubator (model SPX-250B, Shanghai Boxun Medical Biological Instrument Corp., Shanghai, China) [9]. The concentrations of lactic acid, acetic acid, propionic acid, and butyric acid were analyzed via HPLC (Agilent Technologies, Santa Clara, CA, USA) under specified conditions. For chemical composition, DM content was determined by oven-drying at 65 °C for 72 h, CP was analyzed using the Kjeldahl method, and IVDMD was quantified after 48-h incubation with an Ankom DaisyII incubator (ANKOM Technology, Macedon, NY, USA) [8].

### 2.4. Analysis of Nitrogen Fractions

To analyze the nitrogen fractions, 40 mL of the filtrate obtained in Section 2.3 was mixed with 10 mL of a 25% (*v*/*v*) trichloroacetic acid (TCA) solution. The mixture was allowed to stand at room temperature for 1 h to precipitate the true protein. Subsequently, it was centrifuged at 4 °C and 18,000× *g* for 15 min using a refrigerated centrifuge (model 5804R, Eppendorf, Hamburg, Germany). The resulting supernatant, which contained the non-protein nitrogen fractions, was collected for the determination of ammonia nitrogen (NH_3_-N), free amino acid nitrogen (FAA-N), and peptide nitrogen (Peptide-N) according to the method described by [11].

### 2.5. Analysis of Protein Components and Protease Activity

The silage samples were oven-dried in a forced-air oven (model DHG-9203A, Shanghai Shanzhi Instrument Equipment Co., Ltd., Shanghai, China), ground, and passed through a 1-mm sieve. The contents of soluble protein (SOLP) and non-protein nitrogen (NPN) were analyzed according to the method of Licitra et al. [12]. Subsequently, the neutral detergent-insoluble protein (NDIP) and acid detergent-insoluble protein (ADIP) contents were quantified using the sequential detergent extraction procedure described by Sniffen et al. [13]. Based on these measurements, the protein fractions (PA, PB1, PB2, PB3, PC) under the Cornell Net Carbohydrate and Protein System (CNCPS) framework were calculated following the computational methodology established by Sniffen et al. [13]. The activities of carboxypeptidases (CPs), aminopeptidases (APs), and acid protease (AcPr) were determined using commercially available ELISA kits (Shaanxi Biorace Technologies Co., Ltd., Xi’an, China).

### 2.6. Bacterial Community Analysis

#### 2.6.1. Sample Preparation, DNA Extraction, and PCR Amplification

After 60 days of ensiling, silage samples from each treatment were collected and immediately frozen at −80 °C until analysis. For each sample, a 5 g aliquot was used for microbial DNA extraction. Total genomic DNA was extracted using the PowerSoil^®^ DNA Isolation Kit (Mo Bio Laboratories, Carlsbad, CA, USA) following the manufacturer’s instructions. DNA quality was assessed by 1% agarose gel electrophoresis, and concentration and purity were determined using a NanoDrop 2000 spectrophotometer (Thermo Fisher Scientific, Waltham, MA, USA). The V3–V4 hypervariable regions of the bacterial 16S rRNA gene were amplified by polymerase chain reaction (PCR) using the universal primers 338F (5′-ACTCCTACGGGAGGCAGCA-3′) and 806R (5′-GGACTACHVGGGTWTCTAAT-3′). The PCR mixture (20 μL total volume) consisted of 4 μL of 5× TransStart FastPfu buffer, 2 μL of 2.5 mM dNTPs, 0.8 μL of each primer (5 μM), 0.4 μL of TransStart FastPfu DNA polymerase, 10 ng of template DNA, and ddH_2_O. Thermal cycling was performed on an ABI GeneAmp^®^ 9700 instrument (Applied Biosystems, Foster City, CA, USA) under the following conditions: initial denaturation at 95 °C for 3 min; 27 cycles of denaturation at 95 °C for 30 s, annealing at 55 °C for 30 s, and extension at 72 °C for 30 s; followed by a final extension at 72 °C for 10 min. Each sample was amplified in triplicate.

#### 2.6.2. Library Construction and Sequencing

The triplicate PCR amplicons for each sample were pooled and purified. The pooled products were separated on a 2% agarose gel, and the target bands were excised and purified using the AxyPrep DNA Gel Extraction Kit (Axygen Biosciences, Union City, CA, USA). The concentration of the purified amplicons was quantified using a Quantus™ Fluorometer (Promega, Madison, WI, USA). Subsequently, the amplified products from all samples were sent to Shanghai Personal Biotechnology Co., Ltd. (Shanghai, China) for further processing. The service provider constructed the sequencing libraries following the standard Illumina TruSeq 6000 DNA library preparation protocol and performed paired-end (2 × 250 bp) sequencing on an Illumina MiSeq platform (Illumina, San Diego, CA, USA).

### 2.7. Statistical Analysis

Data were summarized using Excel 2021. All statistical analyses were performed using SPSS version 26.0. Prior to analysis, data were tested for normality using the Shapiro–Wilk test and for homogeneity of variances using Levene’s test. One-Way ANOVA was then conducted to evaluate the effects of WGH addition level on the measured variables, with each individual silage bag considered as the experimental unit. When One-Way ANOVA indicated a significant treatment effect (*p* < 0.05), means were compared using Duncan’s (α = 0.05) multiple range test. Linear and quadratic curve estimations were performed using orthogonal polynomial contrasts to examine the dose–response relationships between WGH addition level and silage parameters. Pearson correlation analysis was performed to evaluate the relationships among fermentation parameters, protein fractions, and microbial community indices, and the correlation matrix was visualized using a heatmap constructed with Origin2021. Results are presented as mean values, with *p* < 0.05 considered statistically significant and *p* > 0.05 indicating no significant difference. High-throughput sequencing data of the bacterial community were processed and analyzed on the Genes Cloud online platform (https://www.genescloud.cn).

## 3. Results

### 3.1. Effect of WGH on the Quality of Alfalfa Silage

As the proportion of WGH increased, DM content rose significantly (*p* < 0.05), while CP content showed a slight decreasing trend. The LA, AA, and PA contents of mixed silage decreased, while the pH value increased, with no BA detected. Molds were only detected in the CK. For lactic acid bacteria, although A3 showed the highest numerical value, no significant differences were observed among treatments (*p* > 0.05). Yeast counts in the A3 group were significantly higher than those in the CK and the A1 group (*p* < 0.05), but did not differ significantly from the A2 group. The IVDMD of mixed alfalfa silage was higher than that of the CK, and reached the highest level with the A2 treatment (*p* < 0.05).

As the WGH addition level increased, DM content, pH, and yeast counts increased linearly (*p* < 0.05), while CP, LA, AA, and PA contents decreased linearly (*p* < 0.05). Significant quadratic effects were also detected for DM, CP, pH, LA, AA, and PA (*p* < 0.05) as shown in Table 2.

**Table 2:** Effects of adding walnut green husk on the quality of alfalfa silage.

Items	CK	A1	A2	A3	SEM	Group	Linear	Quadratic
DM (%FM)	28.8 d	34.6 c	37.0 b	38.1 a	1.12	*p* < 0.01	*p* < 0.01	0.01
CP (%DM)	18.4 a	17.2 b	16.8 b	16.4 c	0.74	*p* < 0.01	*p* < 0.01	*p* < 0.01
IVDMD (%DM)	64.6 b	65.2 b	65.9 a	64.7 b	1.69	0.721	0.546	0.367
pH	4.62 c	4.81 b	4.85 b	4.97 a	0.06	*p* < 0.01	*p* < 0.01	*p* < 0.01
LA (%DM)	5.87 a	5.56 b	4.31 bc	3.41 c	0.30	*p* < 0.01	*p* < 0.01	*p* < 0.01
AA (%DM)	1.60 a	1.55 a	1.02 b	0.61 c	0.12	*p* < 0.01	*p* < 0.01	*p* < 0.01
PA (%DM)	0.65 a	0.35 ab	0.33 ab	0.27 b	0.05	*p* < 0.01	*p* < 0.01	*p* < 0.01
BA (%DM)	ND	ND	ND	ND				
LAB (log_10_CFU/g)	7.17 a	7.18 a	7.23 a	7.25 a	0.03	0.753	0.379	0.851
Yeast (log_10_CFU/g)	4.06 c	4.17 b	4.40 a	4.43 a	0.04	*p* < 0.01	*p* < 0.01	0.071
Mold (log_10_CFU/g)	5.65	ND	ND	ND				

Note: Data in the same column with different small letters indicate a significant difference (Duncan; α = 0.05); DM, dry matter; FM, fresh matter; CP, crude protein; IVDMD, in vitro dry matter digestibility; LA, lactic acid; AA, acetic acid; PA, propionic acid; BA, butyric acid; LAB, lactic acid bacteria; CFU, colony forming units. CK: control group, A1: Addition of 80 g/kg walnut green husk, A2: Addition of 120 g/kg walnut green husk, A3: Addition of 160 g/kg walnut green husk. SEM: standard error of the mean (n = 3 per treatment).

### 3.2. Effect of WGH on Nitrogen Distribution of Alfalfa Silage

As shown in Table 3, groups A1, A2, and A3 had significantly lower contents of NPN, Peptide-N, FAA-N, and NH_3_-N than the CK (*p* < 0.05). For NPN, Peptide-N, and FAA-N, the highest WGH inclusion levels (A2 and A3) resulted in similarly low values with no significant differences between them. For NH_3_-N, A2 showed a significantly lower content than A3 (*p* < 0.05). Orthogonal polynomial contrast analysis revealed a significant linear effect of WGH addition on NPN concentration (*p* < 0.05), and significant linear and quadratic effects on Peptide-N, NH_3_-N, and FAA-N (*p* < 0.01).

**Table 3:** Effects of adding walnut green husk on nitrogen component of alfalfa silage.

Items	CK	A1	A2	A3	SEM	Group	Linear	Quadratic
NPN (%/TN)	72.9 a	68.1 b	67.7 b	67.6 b	1.33	0.031	0.014	0.067
Peptide-N (%/TN)	34.2 a	33.6 b	31.1 b	31.8 b	0.36	*p* < 0.01	*p* < 0.01	*p* < 0.01
FAA-N (%/TN)	33.1 a	31.1 b	30.9 c	30.9 c	0.26	*p* < 0.01	*p* < 0.01	*p* < 0.01
NH_3_-N (%/TN)	6.30 a	5.51 b	5.21 b	5.33 c	0.32	*p* < 0.01	*p* < 0.01	*p* < 0.01

Note: Data in the same column with different small letters indicate a significant difference (Duncan; α = 0.05); NPN, non-protein nitrogen; TN, total nitrogen; FAA-N, free amino acid nitrogen; NH_3_-N, ammonia nitrogen. CK: control group, A1:addition of 80 g·kg^−1^ walnut green husk, A2:addition of 120 g·kg^−1^ walnut green husk, A3:addition of 160 g·kg^−1^ walnut green husk. SEM: standard error of the mean (n = 3 per treatment).

### 3.3. Effect of WGH on CNCPS Protein Fractions of Alfalfa Silage

As the proportion of WGH increased, the levels of PB1 and PB2 gradually rose, while NPN, PB3, and PC levels decreased. In groups A1, A2, and A3, PB1 and PB2 were higher than in the CK (*p* < 0.05), whereas NPN, PB3, and PC levels were lower than those in CK (*p* < 0.05). As the proportion of WGH increases, the contents of NPN, PB3, and PC in CNCPS components of silage feed linearly decrease (*p* < 0.05), while the contents of PB1 and PB2 linearly increase (*p* < 0.05). Meanwhile, the addition amount of walnut husk shows a quadratic curve effect on all indicators except NPN in the CNCPS component of the alfalfa mixed silages (*p* < 0.05), as shown in Table 4.

**Table 4:** Effects of adding walnut green husk on CNCPS protein fractions of alfalfa silage.

Items	CK	A1	A2	A3	SEM	Group	Linear	Quadratic
NPN (%/TN)	72.9 a	68.1 b	67.7 b	67.6 b	1.33	0.031	0.014	0.067
PB1 (%/TN)	2.60 b	3.52 a	3.56 a	3.58 a	0.12	*p* < 0.01	*p* < 0.01	*p* < 0.01
PB2 (%/TN)	15.9 b	20.9 a	21.6 a	21.7 a	0.70	*p* < 0.01	*p* < 0.01	*p* < 0.01
PB3 (%/TN)	2.32 a	1.59 b	1.27 c	1.23 c	0.13	*p* < 0.01	*p* < 0.01	*p* < 0.01
PC (%/TN)	6.17 a	5.96 b	5.83 c	5.70 d	0.05	*p* < 0.01	*p* < 0.01	*p* < 0.01

Note: Data in the same column with different small letters indicate a significant difference (Duncan; α = 0.05); NPN, non-protein nitrogen; PB1, rapidly degradable true protein; PB2, intermediately degradable true protein; PB3, slowly degradable true protein; PC, non-degradable protein. All values are expressed as a percentage of total crude protein. CK: control group, A1:addition of 80 g·kg^−1^ walnut green husk, A2:addition of 120 g·kg^−1^ walnut green husk, A3:addition of 160 g·kg^−1^ walnut green husk. SEM: standard error of the mean (n = 3 per treatment).

### 3.4. Effect of WGH on Protease Activity in Alfalfa Silage

**Figure 1:**
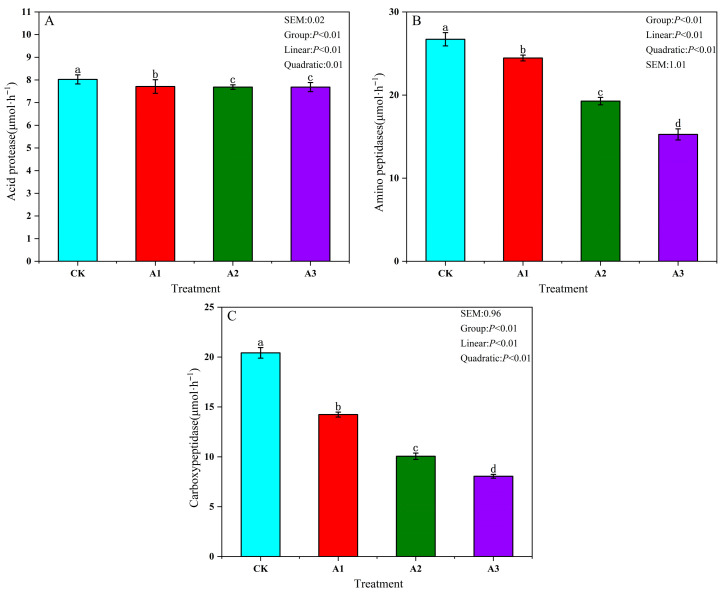
Effects of walnut green husk on protease activity of alfalfa silage. (**A**) Acid protease (AcPr) activity; (**B**) Aminopeptidase (Aps) activity; (**C**) Carboxypeptidase (Cps) activity. Different lowercase letters (a, b, c, d) above the bars indicate significant differences among treatments according to Duncan’s multiple range test at α = 0.05.

Figure 1 shows that in groups A1, A2, and A3, the contents of AcPr, Aps, and Cps were lower than in the CK (*p* < 0.05). As the proportion of WGH addition increased, the activities of Acpr, Aps, and Cps in the protease components of silage feed showed a linear decrease (*p* < 0.05). Meanwhile, the addition amount of WGH had a quadratic curve effect on the activities of Acpr, Aps, and Cps in the protease components of silage feed (*p* < 0.05).

Different lowercase letters above the bars indicate significant differences among treatments according to Duncan’s multiple range test at α = 0.05. CK: control group; A1: addition of 80 g/kg walnut green husk; A2: addition of 120 g/kg walnut green husk; A3: addition of 160 g/kg walnut green husk. SEM: standard error of the mean (n = 3 per treatment).

### 3.5. Effects of WGH on Bacterial Community Structure of Alfalfa Silage

**Figure 2:**
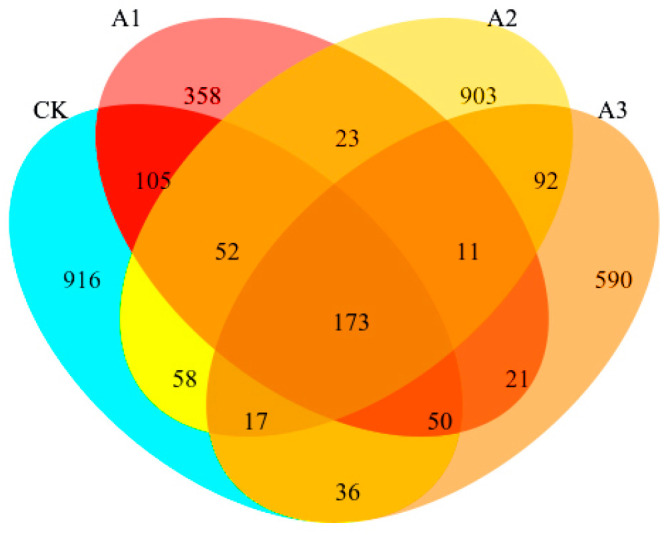
Effect of adding Walnut green husk on the distribution of OTU in alfalfa silage. CK: control group, A1: addition of 80 g/kg walnut green husk, A2: addition of 120 g/kg walnut green husk, A3: addition of 160 g/kg walnut green husk.

**Figure 3:**
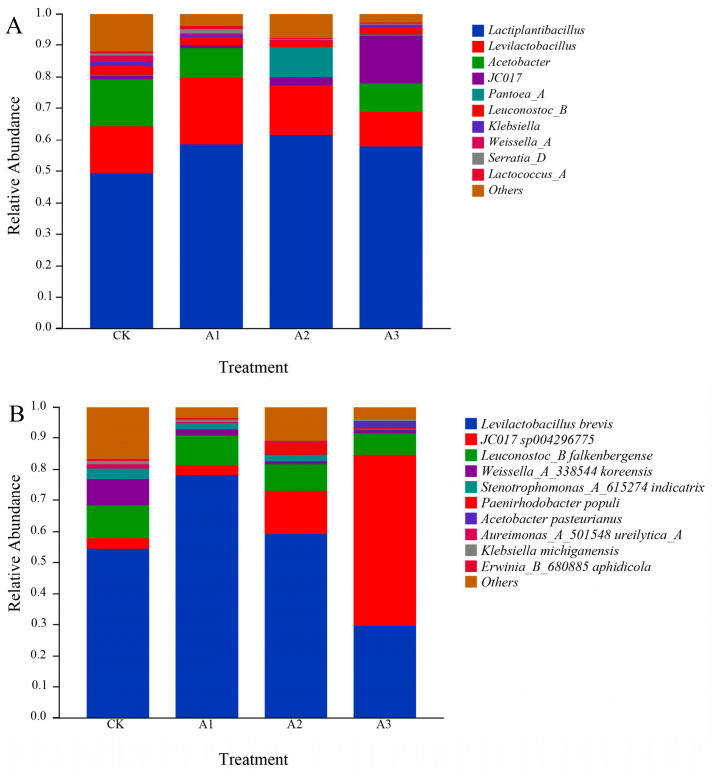
Effect of adding Walnut green husk on the bacterial community structure in alfalfa silage. (**A**) genus level; (**B**) species level CK: control group, A1: addition of 80 g/kg walnut green husk, A2: addition of 120 g/kg walnut green husk, A3: addition of 160 g/kg walnut green husk.

**Figure 4:**
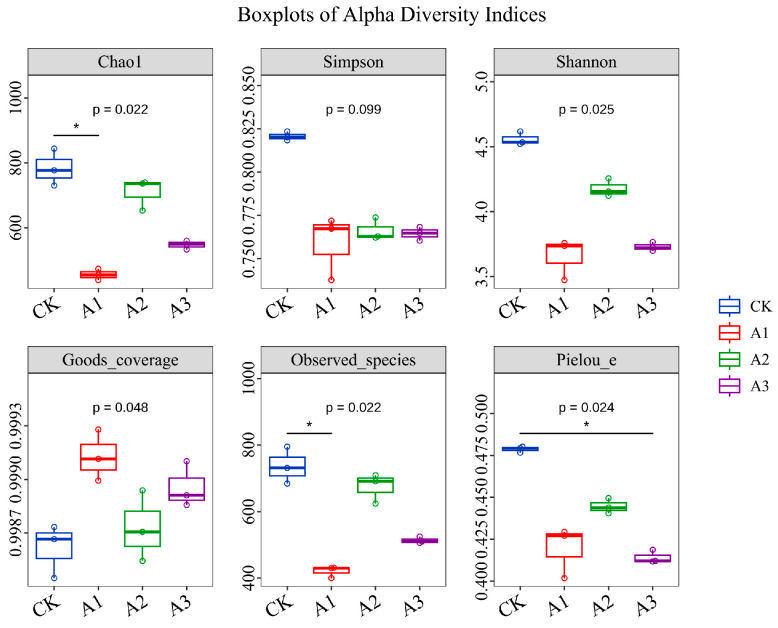
Effects of adding walnut green husk on α diversity index of Alfalfa silage. CK: control group, A1: addition of 80 g/kg walnut green husk, A2: addition of 120 g/kg walnut green husk, A3: addition of 160 g/kg walnut green husk. * Indicates significant difference compared to the CK at *p* < 0.05.

**Figure 5:**
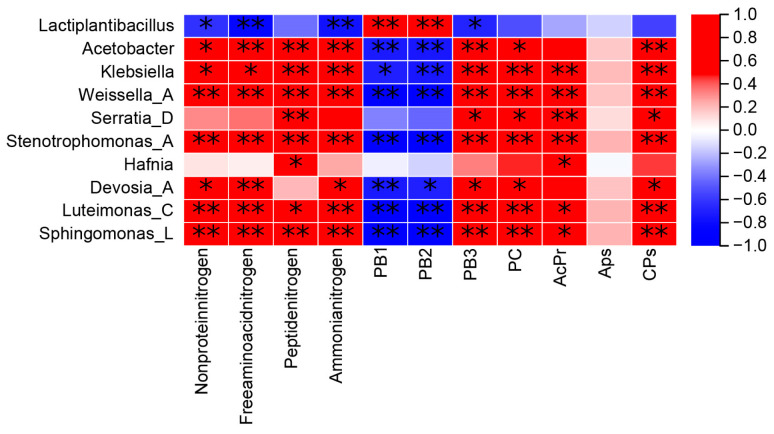
Pearson correlation heat map between bacterial community genus and protein factors * and ** mean significant difference with a *p*-value of <0.05 and 0.01, respectively. PB1, Rapidly Degradable Protein; PB2, Moderately Degradable Protein; PB3, Slowly Degradable Protein PC, Unavailable Protein; AcPr, acid protease; APs, aminopeptidases; CPs, carboxypeptidases.

Figure 2 shows that the number of OTUs in the bacterial community after adding WGH to alfalfa silage decreased compared to the control group (CK). The OTU counts for the treatment groups were CK, A2, A3, and A1, listed from highest to lowest. Figure 3A (genus level) and Figure 3B (species level) illustrate the relative abundance changes in the top 10 bacterial communities in alfalfa silage after the addition of WGH. With increasing WGH proportion, the relative abundances of *Lactiplantibacillus* and *Levilactobacillus* were higher in the treatment groups than in the CK. At the species level, the relative abundances of *Stenotrophomonas_A_615274 indicatrix*, *Klebsiella michiganensis*, and *Erwinia_B_680885 aphidicola* decreased with higher levels of WGH, while *Paenirhodobacter populi* increased. The α diversity results (Figure 4) indicate that after adding WGH, Compared with the CK, the Chao1 and Observed_species indices were significantly lower in the A1 group (*p* < 0.05), and the Pielou_e index was significantly lower in the A3 group (*p* < 0.05). No significant differences were detected among treatments for the Simpson or Shannon indices, and Goods_coverage remained above 99% in all groups with no significant differences. Pearson correlation analysis assessed the relationships between the top 10 bacterial species relative abundances and Protein degradation factors, as depicted in Figure 5 shows that Lactiplantibacillus was extremely significantly positively correlated with PB1 and PB2 (*p* < 0.01), and exhibited negative correlations with FAA-N and NH_3_-N (*p* < 0.01) as well as with NPN and PB3 (*p* < 0.05). The other nine marker microbial genera show a negative correlation with PB1 and PB2, while showing a positive correlation with other indicators.

## 4. Discussion

### 4.1. Effect of WGH Addition on the Quality of Alfalfa Silage

The relatively high WSC (7.54%) and tannin (12.24 mg/g) contents in WGH may have contributed to the improved fermentation quality and reduced protein degradation observed in WGH-treated silages, possibly by providing additional fermentable substrates and inhibiting proteolytic activity. The high moisture content of fresh alfalfa (generally >75%) is detrimental to silage quality, as it promotes clostridial activity and influences microbial population dynamics and fermentation rates [14]. Therefore, alfalfa is typically wilted or blended with high dry matter materials prior to ensiling. In this study, the inclusion of WGH increased the DM content of the mixed silages, with A1 (34.55%) and A2 (37.01%) falling within the recommended range of 30–35% for ensiled materials, while A3 (38.12%) exceeded this range [15]. pH is a critical indicator for evaluating silage quality [16]. Excessively high pH favors the growth of undesirable microorganisms such as Clostridium, impairing fermentation, while excessively low pH may inhibit LAB and compromise silage preservation [17]. In the present experiment, the pH of all silages ranged from 4.62 to 4.97, which falls within the acceptable range for alfalfa silage (4.30–5.00) and was not associated with any off-odors [18]. High-quality silage should typically contain >3% LA, 1–4% AA, and minimal BA on a DM basis [19]. All treatment groups in this study met these criteria. The lower organic acid content observed in the WGH-supplemented groups compared with alfalfa-only silage further suggests that tannin-rich materials can inhibit the proliferation of acid-producing bacteria [20]. Notably, mold was detected only in the alfalfa-only silage, implying that WGH addition provided sufficient fermentable substrates under anaerobic conditions, thereby suppressing mold development and improving the overall fermentation quality of alfalfa silage. IVDMD is a key measure of feed conversion efficiency in ruminants, reflecting the ease with which feed is degraded [15]. Group A2 significantly enhanced the IVDMD of alfalfa silage. Alternatively, condensed tannins present in WGH could inhibit harmful microorganisms, reduce proteolysis and leach of cellular contents, and better preserve nutrients, ultimately leading to higher IVDMD.

### 4.2. Effect of WGH Addition on Nitrogen Fraction of Alfalfa Silage

Protein degradation, driven by plant and microbial enzymes during alfalfa ensiling, converts true protein into NPN. This process can account for 44–87% of total nitrogen, substantially reducing protein utilization [21]. In the present study, the addition of WGH induced a linear decrease in NPN content, indicating suppressed protein hydrolysis and improved utilization efficiency. This effect may be associated with two potential mechanisms. One possibility is that the high DM content of WGH contributed to the inhibition of protease activity, thereby limiting protein conversion to NPN, although the individual roles of these factors require further investigation [22]; second, the tannins in WGH may bind to proteins, increasing the proportion of true protein that resists degradation [23]. The NPN fraction comprises NH_3_-N, FAA-N, and Peptide-N, all of which influence protein availability. In our experiment, the contents of these three components progressively decreased with WGH addition, with the A2 group showing the lowest levels of NH_3_-N and Peptide-N. This reduction is likely related to the tannin-rich profile of WGH, which may bind to and inhibit key proteases (AcPr, APs, and CPs), resulting in reduced production of FAA-N and Peptide-N as hydrolysis products. Consequently, the subsequent deamination of FAA-N by microbial proteases was also diminished, leading to lower NH_3_-N accumulation. The observed reduction in NPN content and the increase in PB1 with WGH addition are consistent with previous studies reporting improved nitrogen preservation in alfalfa silage treated with tannin-rich additives [8,24].

Within the CNCPS framework, the PB fraction is categorized by degradation rate: PB1, PB2, and PB3 degradable. PB1 and PB2 serve as crucial nitrogen sources for rumen microbial protein synthesis [25]. In this study, increasing WGH inclusion significantly raised the contents of PB1 and PB2 while gradually lowering PB3. This shift suggests that WGH may modify fermentation substrates, inhibit plant protease activity and clostridial fermentation, thereby enhancing the soluble, readily available protein pool (PB1 and PB2) in the rumen. Conversely, the decline in PB3 the less beneficially utilized fraction often associated with rumen undegraded protein (RUP) implies a reduction in the proportion of protein that escapes rumen fermentation. This shift likely increases the proportion of protein absorbed post ruminal, ultimately improving the overall protein utilization efficiency of the silage. The underlying mechanism may involve an optimization of the protein molecular structure in alfalfa silage upon WGH addition, which in turn affects protease accessibility and slows degradation [26].

### 4.3. Effects of Adding WGH on Bacterial Community of Alfalfa Silage

The fermentation of silage involves complex and dynamic microbial changes, where the composition and quantity of microorganisms directly determine the quality of the final product. In this experiment, the Goods_coverage index for all samples approached 1, indicating that the sequencing depth sufficiently represented the in situ microbial community. In some studies, improved silage fermentation has been linked to reduced microbial diversity, as the ensiling process selectively shapes community structure [27,28]. Consistent with this, it could be hypothesized that the addition of WGH may contribute to a more rapid establishment of anaerobic conditions during ensiling, though this hypothesis requires confirmation through direct measurement of oxygen dynamics in future studies. Concurrently, WGH treatment increased the relative abundance of beneficial genera such as *Lactiplantibacillus* and *Levilactobacillus*, which are known to improve silage quality and prevent spoilage [29]. At the species level, *Levilactobacillus brevis* became the most dominant species, potentially inhibiting harmful bacteria and reducing microbial competition [30]. Furthermore, *Lactiplantibacillus* showed a highly significant negative correlation with NH_3_-N and FAA-N, suggesting a close relationship between this genus and nitrogen metabolism during ensiling, thereby helping to curb protein degradation and the deamination of amino acids. In summary, WGH addition favorably alters the bacterial community by enhancing beneficial lactic acid bacteria and potentially inhibiting proteolysis, collectively contributing to improved fermentation quality and protein preservation in alfalfa silage.

## 5. Conclusions

Adding WGH to alfalfa silage was associated with reduced organic acid and NH_3_-N contents and increased LAB counts, suggesting a potential improvement in fermentation quality, particularly in reducing undesirable fermentation products. Furthermore, this treatment was correlated with higher DM content, improved nutritional quality, increased IVDMD, and reduced protein degradation, indicating a possible link between WGH addition and plant protease activity during ensiling. It also has a positive effect on protein fractions such as NPN, PB2, and PB3 during alfalfa silage fermentation. In addition, with the increase in WGH addition ratio, it reduces the diversity of bacterial communities, increases the relative abundance of beneficial bacteria *Lactiplantibacillus* and *Levilactobacillus*, and decreases the relative abundance of *Klebsiella* and *Serratia_D*, effectively increasing the abundance of beneficial bacterial communities. Overall, considering the comprehensive effects on fermentation quality, protein fractions, and digestibility, the inclusion of 120 g/kg WGH appeared to be the most suitable among the tested levels.

## Figures and Tables

**Table 1:** Nutrient composition of raw materials in silage (on a DM basis, %).

Items	Alfalfa	WGH
DM (%)	29.5 ± 0.32	92.3 ± 0.41
Ash (%)	11.3 ± 0.21	17.2 ± 0.18
CP (%)	18.4 ± 0.25	13.2 ± 0.19
WSC (%)	2.51 ± 0.08	7.54 ± 0.12
NDF (%)	36.8 ± 0.54	50.3 ± 0.62
ADF (%)	30.2 ± 0.48	47.7 ± 0.55
Tannin(mg/g)	—	12.2 ± 0.15

Note: WGH, Walnut green husk; DM, dry matter; CP, crude protein; WSC, water-soluble carbohydrate; NDF, neutral detergent fiber; ADF, acid detergent fiber. Values are presented as mean ± SD (n = 3).

## Data Availability

The datasets presented in this study can be found in online repositories. The names of the repository/repositories and accession number(s) can be found at: https://www.ncbi.nlm.nih.gov/bioproject/PRJNA1400221 (accessed on 8 January 2026).
